# Cardiovascular hospitalization dynamics in Iran’s second-largest city: A spatial and temporal perspective

**DOI:** 10.1371/journal.pone.0352424

**Published:** 2026-07-06

**Authors:** Shahab MohammadEbrahimi, Tahereh Samimi, Mohammad Dehghan, Munazza Fatima, Elahe Zare, Atieh Sedghian, Saeid Eslami, Behzad Kiani

**Affiliations:** 1 Department of Medical Informatics, School of Medicine, Mashhad University of Medical Sciences, Mashhad, Iran; 2 School of Population and Public Health, University of British Columbia, Vancouver, British Columbia, Canada; 3 Department of Medical Informatics, Urmia University of Medical Sciences, Urmia, Iran; 4 Department of Anesthesiology and Critical Care, Tehran University of Medical Sciences, Tehran, Iran; 5 Department of Geography and Geoinformatics, The Islamia University Bahawalpur, Bahawalpur, Pakistan; 6 Cardiovascular Research Center, Shahid Sadoughi University of Medical Sciences, Yazd, Iran; 7 Frazer Institute, Faculty of Health, Medicine and Behavioural Sciences, The University of Queensland, Brisbane, Queensland, Australia; Maragheh University of Medical Sciences, IRAN, ISLAMIC REPUBLIC OF

## Abstract

**Introduction:**

Cardiovascular disease (CVD) is a leading cause of mortality worldwide, with an especially high burden in developing countries such as Iran. Understanding spatial disparities and temporal trends in CVD hospitalizations can guide targeted public health interventions in high-risk regions.

**Methods:**

Data was sourced from Mashhad University of Medical Sciences for five years (2016–2020) and included CVD cases classified by ICD-10 codes, excluding records with non-resident status or incomplete addresses. Temporal trends were assessed through a monthly classical decomposition, ARIMA modeling for forecasting, and Joinpoint regression (JR) to detect shifts over time. Spatial analyses included calculating relative risks, examining spatial autocorrelation, identifying hotspots, and applying flexible spatial scan statistics (FSSS) to detect clusters with irregular shapes.

**Results:**

The study included 52,132 CVD cases, with a median age of 64 years (IQR: 53_76) and a male predominance (54.44%). Temporal analysis showed significant fluctuations, with the highest hospitalization rate in 2019 and the seasonal pattern was repeated annually. ARIMA(0,0,0)(0,0,1)_12_ modeling revealed a seasonal moving average of −0.722 and a mean of 844.95 hospitalizations/month, capturing seasonal fluctuations with a stable trend reflecting cardiovascular health risks. The JR analysis showed a rising hospitalization trend with a monthly percent change of 1.69 [95% CI: 1.44_2.29; *p* = 0.001] for males and 1.99 [95% CI: 1.73_2.68; *p* = 0.001] for females, followed by a sharp decline after November 2019. After a positive spatial autocorrelation (Global Moran’s *I* of 0.176 (*p* < 0.001)), hot spot classification revealed central Mashhad as a high-risk zone with concentrated clusters of CVD hospitalizations. The Flexible Spatial Scan Statistics (FSSS) analysis indicated that the Most Likely Cluster (MLC) was situated in the city center, presenting an approximate 10-fold relative risk [RR: 9.804, LLR: 2328.3, *p* < 0.001].

**Conclusions:**

This study provides the first extensive spatial and temporal analysis of CVD hospitalizations in Mashhad, underscoring critical high-risk areas in need of focused public health interventions. Addressing these spatial disparities can significantly improve cardiovascular health outcomes and bolster resilience within the local populations.

## 1. Introduction

Cardiovascular disease (CVD) stands out as a prominent type of non-communicable disease (NCD) worldwide [1]. According to the World Health Organization (WHO), NCDs account for approximately 74% of global deaths, with CVD holding the highest position (32%) [2] and taking an estimated 17.7 million lives annually [3]. Despite specific advancements in the prevention and treatment of CVD in developed nations, its burden remains substantial in developing countries, contributing to nearly 60% of the global disease burden [4]. The global burden of CVD has increased dramatically, with deaths rising by 49% compared to 1990 [5]. This epidemiological transition is particularly pronounced in low- and middle-income countries (LMICs), where CVD mortality rates are disproportionately higher compared to high-income countries, with more than 80% of CVD deaths occurring in these settings [4,6]. In Iran, as a developing country, CVDs also present a significant cause of both mortality and morbidity, constituting 46% of all deaths and 20%−23% of the disease burden [7]. A substantial proportion of the Iranian population faces moderate to high risk of cardiovascular events within the next decade, with urgent preventive plans needed at the national level [8].

A 20-year US study found that 44% of the decline in cardiovascular deaths was due to modifiable factors [9]. Modifiable risk factors, which include lifestyle and environmental aspects that can be changed to reduce the risk of developing CVD [10], significantly contribute to spatial variations in CVD prevalence worldwide. For example, research has shown that residing in underprivileged neighborhoods is linked to a higher prevalence of CVD [11]. Moreover, socioeconomic determinants such as income, education, and access to healthcare play a crucial role in these spatial disparities [12]. Therefore, understanding geographical patterns and environmental influences can offer key insights into health problems.

The application of spatiotemporal analysis to CVD research has emerged as a critical methodological approach for understanding disease patterns and informing public health interventions. Spatial epidemiology techniques, including Geographic Information Systems (GIS) and Bayesian spatial modeling, provide powerful tools for analyzing CVD distribution and identifying high-risk areas [13,14]. Studies utilizing spatiotemporal analysis have identified distinct patterns in CVD hospitalization. The application of space-time scan statistics and spatial autocorrelation analysis has proven particularly valuable in identifying high-risk areas and periods for cardiovascular events [15].

A 2018 review identified a correlation between CVD prevalence and socioeconomic determinants, hypothesizing that environmental factors may also contribute to the spatial distribution of CVD [16]. Another study in Sweden found that CVD cases were geographically clustered, with significant regional disparities and gender variations observed [17]. Similarly, distinct spatial patterns have been reported in different regions, underscoring the role of place-based factors in CVD risk.

In the context of Iran, a national study identified clusters of CVD across six provinces, revealing a similar pattern between genders and linking environmental risk factors to CVD health outcomes [18]. Another spatiotemporal analysis of CVD mortality in Iran from 2017 to 2019 identified a rising mortality trend, especially among elderly males [19]. Mashhad, Iran’s second-largest city with approximately 3 million inhabitants and receiving around 25 million tourists annually [13], represents a unique urban environment for cardiovascular health research. The urban context of Mashhad presents particular challenges for cardiovascular health, with studies documenting the impact of environmental factors on cardiovascular mortality [20]. A spatial analysis conducted in Mashhad, the focal point of our study, revealed a significant association between urban green space and the occurrence of CVD in women, indicating that greater access to green space correlated with a reduced risk of CVD. The study specifically focused on factors affecting women’s access to and proximity to these green spaces, while excluding considerations of men’s gender and other built environment factors beyond green spaces. [21]. Another study conducted in this city highlighted that socioeconomic factors, such as income and education, were linked to health outcomes, with lower socioeconomic status associated with a higher prevalence of chronic diseases. Notably, this study addressed a range of chronic diseases beyond just CVD [12].

Although the widespread burden of CVD and spatial disparities are well recognized globally and nationally, comprehensive spatiotemporal analyses focusing specifically on cardiovascular hospitalization patterns in Iranian urban centers—particularly in large metropolitan areas such as Mashhad—remain limited. Current literature largely focuses on mortality or prevalence at broad provincial or national levels, missing the granular insights provided by small-area spatial and temporal studies of hospitalization dynamics. These detailed local analyses are essential because small-area variations in disease burden and healthcare utilization can differ substantially from national averages, influencing healthcare resource planning and targeted interventions. Accordingly, this study addresses this critical gap by utilizing a comprehensive hospitalization dataset and applying advanced geospatial and statistical methods to analyze cardiovascular hospitalization dynamics in Mashhad. By investigating the spatiotemporal patterns our work aims to provide actionable insights for healthcare planning, resource allocation, and tailored public health strategies within Iranian urban contexts. Additionally, it contributes valuable region-specific evidence to the broader field of cardiovascular spatial epidemiology, enhancing our understanding of CVD dynamics in diverse urban settings.

## 2. Materials and methods

### 2.1. Study area

Mashhad, located in the northeastern region of Iran within Razavi-Khorasan province, illustrated in inset maps in Appendix 1, with a population of 3.1 million according to the 2016 census, making it the second largest urban center in Iran. From a geographical perspective, Mashhad is divided into 1,301 census tracts, which serve as the most granular unit of analysis in this study. This level of detail enhances the accuracy and specificity of our results.

### 2.2. Data acquisition and preparation

#### 2.2.1. CVD hospitalization data.

The data pertaining to CVD hospitalizations, from January 1, 2016, to December 31, 2020, was gathered from the hospital information systems (HIS) administered by Mashhad University of Medical Sciences. The data was accessed for research purposes on 20/02/2021. The HIS used in this study covers all public hospitals affiliated with Mashhad University of Medical Sciences, with diagnoses manually recorded using ICD-10 codes and subjected to routine clinical and administrative auditing. The dataset comprises hospitalizations for a range of cardiovascular disorders, classified by international classification of diseases, tenth revision (ICD-10) codes, including ischemic heart disease (I20-I25), atrial fibrillation (I48), heart failure (I50), ischemic stroke (I63, I65-I67, G45, G46), and atherosclerotic disease (I70-I77.1). Each hospitalization record included a unique national identifier, enabling identification of repeated admissions. To avoid risk inflation with multiple hospitalizations and to preserve spatial comparability, duplicated admissions for the same individual were removed, and only the first recorded CVD hospitalization during the study period was retained for analysis.

#### 2.2.2. Data preprocessing and geocoding.

After acquiring the CVD hospitalization data, a preprocessing step was performed on the address field, since patient information was manually recorded at the time of admission. Notably, as Mashhad stands out as a prominent religious pilgrimage destination and tourist hub in Iran, drawing over 20 million visitors annually [22], records indicating addresses at hotels and inns were eliminated from the dataset. Additionally, all entries without address information were removed, as well as those representing residences outside the urban center of Mashhad. Finally, the dataset utilized for patient-related analysis comprised 52,132 refined records. Given the absence of geographic coordinates for patients’ residences, the utilization of the Google My Map service facilitated the geocoding of patient locations. To protect individuals’ confidentiality, a jittering technique introducing a 100-meter variance was applied to the geocoded points.

### 2.3. Temporal analysis

To examine temporal patterns in CVD hospitalizations, we used two complementary methods: Time Series Decomposition with ARIMA Modeling and Joinpoint Regression. ARIMA helped us capture overall trends and seasonal patterns for forecasting, while Joinpoint Regression detected specific shifts in trends over time and allowed for sex-based comparisons. This combined approach provides a robust analysis, capturing both general patterns and distinct temporal changes.

#### 2.3.1. Time series with ARIMA modeling.

In this study, we conducted a time series analysis on CVD hospitalization data to identify trends and seasonal patterns. The data was classically decomposed into its trend, seasonal, and residual components (noise), facilitating a clearer understanding of the underlying behaviors. We employed an Auto-Regressive Integrated Moving Average (ARIMA) model, specifically ARIMA(0,0,0)(0,0,1)_12_, which includes one seasonal moving average term and a seasonal period of 12 months for forecasting. The model was fitted using maximum likelihood estimation, and diagnostic checks were performed to evaluate residual autocorrelation and normality. Forecast extending beyond the observed period was generated, accompanied by confidence intervals to quantify prediction uncertainty [23]. Given the descriptive and exploratory objective of the time series analysis, formal stationarity testing was not undertaken. The selected seasonal ARIMA specification with no differencing (d = 0) was chosen to summarize observed temporal patterns and seasonality rather than to infer long-term stochastic properties of the series.

#### 2.3.2. Joinpoint regression modeling.

The Joinpoint regression program (2017) [24] was used to investigate trends in CVD hospitalization rates from 2016 to 2020. The primary objective was to identify significant shifts, called Joinpoint, in overall admission rates by constructing separate regression models for male and female. These models facilitated the detection of notable increases or decreases in hospitalization patterns. This analytical method also allowed for the calculation of the monthly percent change (MPC) for each identified trend, quantifying the rate of change in hospitalization over time [25]. Joinpoint models were fitted using a log-linear regression framework with hospitalization rates as the dependent variable and time as the independent variable. A Poisson variance structure was assumed, consistent with count-based rate data, and statistical significance of Joinpoint was assessed using permutation tests implemented in the Joinpoint Regression Program.

### 2.4. Spatial statistical analysis

To reduce instability arising from small population sizes in some census tracts, analyses were conducted using standardized incidence ratios (SIRs), which account for the underlying population at risk and expected case counts. The SIR adjusts for differences in population structure, facilitating a fair comparison of CVD risk across regions. Given that population sizes vary by census area (SD = 2,680) and there was a global spatial autocorrelation for CVD hospitalization rates (Global Moran’s Index = 0.176, *p* < 0.001), we used SIR rate to show the smooth pattern of CVD hospitalization.

After employing Global Moran’s *I* to evaluate overall spatial autocorrelation and determine whether hospitalization rates displayed non-random spatial patterns across the study area [26], we utilized two complementary spatial analysis techniques to examine the distribution and clustering of CVD hospitalizations: Getis-Ord Gi* and flexible spatial scan statistics (FSSS). The Getis-Ord Gi* hot spot analysis pinpointed specific areas with significantly high or low hospitalization rates, offering valuable insights into spatial clustering [27]. Meanwhile, FSSS identified irregularly shaped clusters without fixed boundaries, allowing for a more nuanced detection of high-risk areas that may require further investigation [28].

#### 2.4.1. Standardized incidence ratios (SIR).

The SIR [29] served as the input variable for the spatial analysis in this study, calculated for each area as the ratio of observed cases ($Y_{i}$) to expected cases ($E_{i}$). The expected counts reflect the total number of events that would be anticipated if the observed population exhibited the same behavior as the reference or standard population.


$$\begin{array}{c} {SIR}_{i}=\frac{Y_{i}}{E_{i}} \end{array}$$ (1)

The expected counts ($E_{i}$) are calculated as follows, where $\overset{\left(s\right)}{\underset{j}{r}}$ is the rate in stratum *j* (population data for census tracts, ranging from 1 to 1,301, sourced from the 2016 census) of the standard population, and $n_{j}$ denotes the population in stratum *j* of the area. Population denominators for crude rates and expected counts were obtained from the 2016 national census conducted by the Statistical Center of Iran and were applied consistently across the study period due to the absence of annual small-area population estimates.


$$\begin{array}{c} E_{i}\;=\sum_{j=\text{1}}^{m}\;\overset{\left(s\right)}{\underset{j}{r}}*\;n_{j} \end{array}$$ (2)

#### 2.4.2. Spatial autocorrelation and hotspot analysis.

The Getis-Ord Gi* hotspot analysis identified localized clusters by comparing observed values to expected values. High Gi* values indicate hotspots with significantly elevated rates, while low Gi* values indicate cold spots with lower-than-expected rates [27].

#### 2.4.3. Flexible spatial scan statistics.

We adopted an approach based on Tango and Takahashi’s FSSS [28] to detect spatial clusters of CVD with adaptable shapes, unlike the traditional method proposed by Kulldorff (1997) [30], which relies on circular or elliptical scanning windows. This flexibility allows for the identification of clusters that reflect the underlying spatial patterns of disease occurrence. Statistical significance was evaluated using a log-likelihood ratio (LLR) test, which compares the likelihood of the observed number of cases within candidate clusters to that expected outside the clusters under a Poisson assumption. Clusters with significantly elevated or reduced relative risk (RR) were identified, and secondary clusters (SCs) were defined as those not overlapping with clusters exhibiting higher LLR values [31]. The LLR, in a Poisson distribution, assesses the likelihood under two hypotheses: the null hypothesis, which posits no cluster exists, and the alternative hypothesis, which indicates the presence of a significant cluster.

### 2.5. Software

All analyses, including descriptive, non-spatial, and spatial tests, were two-sided with a significance level of *p* < 0.05. The authors conducted all analyses and created maps using R (version 4.3.3), employing packages such as *sf* [32], *spdep* [33], and *rflexscan* [34] for spatial analysis. For the time series analysis, the primary packages utilized were *stats* and *forecast* [35].

### 2.6. Ethics statement

The study was approved by the ethics committee of Mashhad University of Medical Sciences, Mashhad, Iran, with the reference number IR.MUMS.MEDICAL.REC.1399.422. We confirm that all methods were conducted in compliance with the relevant guidelines and regulations. The data used in the analysis were fully anonymized; consequently, the ethics committee of Mashhad University of Medical Sciences, Mashhad, Iran, waived the requirement for obtaining informed consent from participants.

## 3. Results

### 3.1. Descriptive analysis

A total of 52,132 confirmed CVD hospitalizations were included in the analysis. Of these, 28,379 (54.44%) occurred among males. The median age was 64 years (inter-quartile range [IQR]: 53_76 years). The median length of hospital stay (LOS) was 2 days (IQR: 1_5 days). During the study period, 4,907 deaths were recorded, corresponding to an in-hospital mortality proportion of 9.41%. A summary of the included records, which encompasses cases with missing or non-residential addresses, as well as the proportions of sex and death, along with the median age and LOS, is presented in Table 1.

**Table 1:** Characteristics of CVD hospitalization in Mashhad, Iran, Jan 2016 to Dec 2020.

Characteristics	CVD patients
**Initial data (%)**	**203,420 (100)**
Assigned to the city of Mashhad	54,147 (26.61)
*Within the city borders (Geocoded)*	*52,132 (96.27)*
*Outside the city borders (Geocoded)*	*2015 (3.72)*
Completely out of the study area or incomplete address	149,273 (73.39)
**Sex (%)**	**52,132 (100)**
Male	28,379 (54.44)
Female	23,753 (45.56)
**Death (%)**	**4907 (9.41)**
Male	2726 (55.55)
Female	2181 (44.45)
**Median age (IQR)**	**64.0 (53**–**76 years)**
**Median Length of Stay (IQR)**	**2.0 (1**–**5 days)**

CVD: Cardiovascular disease, IQR: Inter-quartile range.

### 3.2. Temporal patterns

#### 3.2.1. Overall trend.

**Fig 1:**
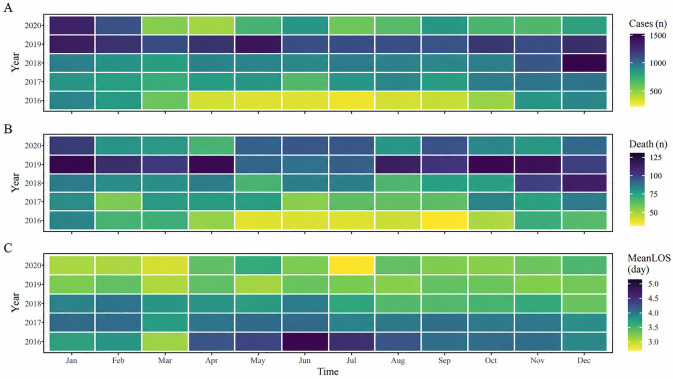
Heat plots showing the monthly frequency of A: cardiovascular disease (CVD) hospitalization (number of cases), B: in-hospital deaths (number of deaths), and C: the average length of stay (days) in the city of Mashhad from 2016 to 2020. Color intensity reflects the magnitude of each variable for a given month and year and does not represent correlation or association measures.

Monthly CVD hospitalization varied over the study period, with the lowest counts observed between April and September 2016. In contrast, 2019 exhibited the highest overall hospitalization counts (Fig 1-A). The number of recorded deaths followed a similar temporal pattern, with the highest values also observed in 2019 (Fig 1-B). During the second wave of the COVID-19 pandemic (May–July 2020), hospitalization counts were lower than in 2019, while the number of deaths remained elevated. The average length of hospital stay was longest in 2016, particularly between April and August (Fig 1-C). Periods with higher hospitalization volumes coincided with shorter average LOS, whereas periods with lower hospitalization volumes coincided with longer LOS.

#### 3.2.2. Time series decomposition and ARIMA forecast.

**Fig 2:**
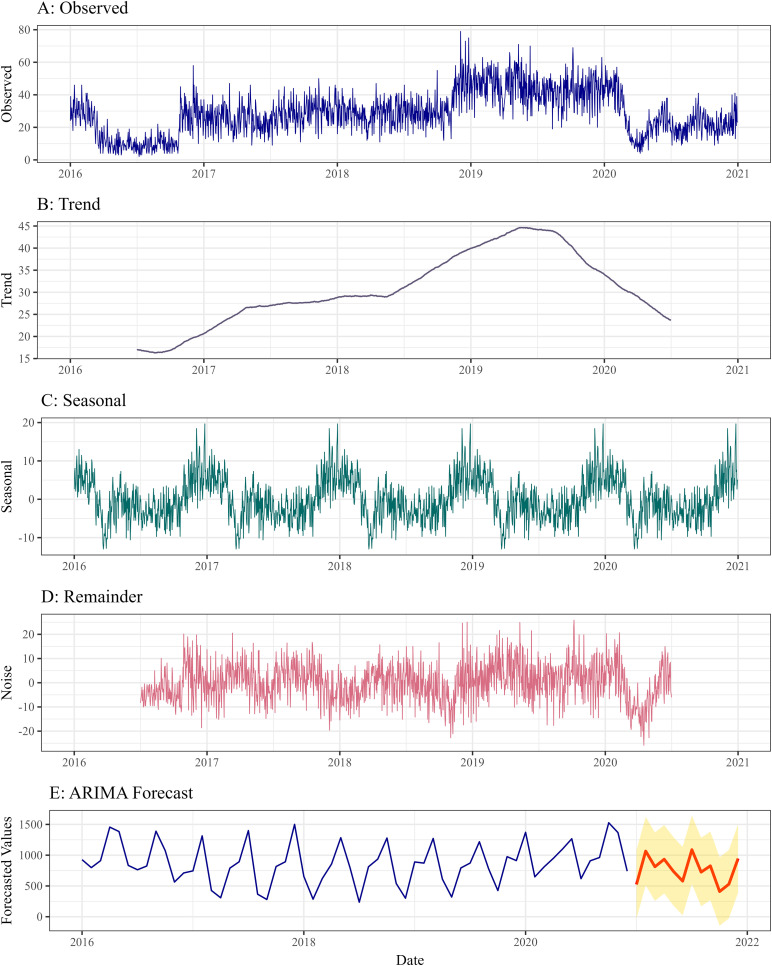
Time series decomposition and ARIMA forecasting of five years of cardiovascular hospitalization data for Mashhad city.

Time series decomposition of monthly CVD hospitalizations demonstrated a long-term trend, a repeating annual seasonal component, and a residual component reflecting irregular variation (Fig 2). The trend increased from 2016, peaked in 2019, and declined thereafter. Seasonal effects exhibited a 12-month periodicity.

The ARIMA(0,0,0)(0,0,1)_12_ model was fitted to the monthly hospitalization series. The seasonal moving average parameter was 0.722 (SE = 0.245, *p* = 0.003). The estimated mean monthly hospitalization count was 844.95 (SE = 19.39, *p* < 0.001). Model diagnostics yielded a variance of 79,537, a log-likelihood of −426.94, AIC = 859.87, and BIC = 866.15. Forecasts through 2022 indicated stabilization around the estimated mean, with widening confidence intervals over time.

#### 3.2.3. Multiple joinpoint model.

Joinpoint regression identified one statistically significant change point in November 2019 for both sexes (Table 2). From January 2016 to November 2019, CVD hospitalization rates increased, with a monthly percent change (MPC) of 1.69 [95% CI: 1.44–2.29; *p* = 0.001] for males and 1.99 [95% CI: 1.73–2.68; *p* = 0.001] for females. However, from November 2019 to December 2020, hospitalization rates declined, with an MPCs of −6.04 [95% CI: −9.53 to −4.34; *p* = 0.001] for males and −6.49 [95% CI: −10.26 to −4.70; *p* = 0.001] for females.

**Table 2:** The sex-adjusted Joinpoint Regression model for cardiovascular disease (CVD) admission rates (per 10,000) in the city of Mashhad, 2016-2020.

Cohort	Segment	Lower Endpoint	Upper Endpoint	MPC	Lower CI	Upper CI	Test Statistic (t)	p-Value
Male – 1 Joinpoint	1	Jan 2016	Nov 2019	1.689	1.441	2.293	7.857	0.001
Male – 1 Joinpoint	2	Nov 2019	Dec 2020	−6.036	−9.527	−4.338	−4.991	0.001
Female – 1 Joinpoint	1	Jan 2016	Nov 2019	1.986	1.734	2.681	8.353	0.001
Female – 1 Joinpoint	2	Nov 2019	Dec 2020	−6.487	−10.263	−4.695	−4.887	0.001

MPC: Monthly percent change, CI: Confidence interval.

### 3.3. Spatial patterns

#### 3.3.1. Spatial heterogeneity.

**Fig 3:**
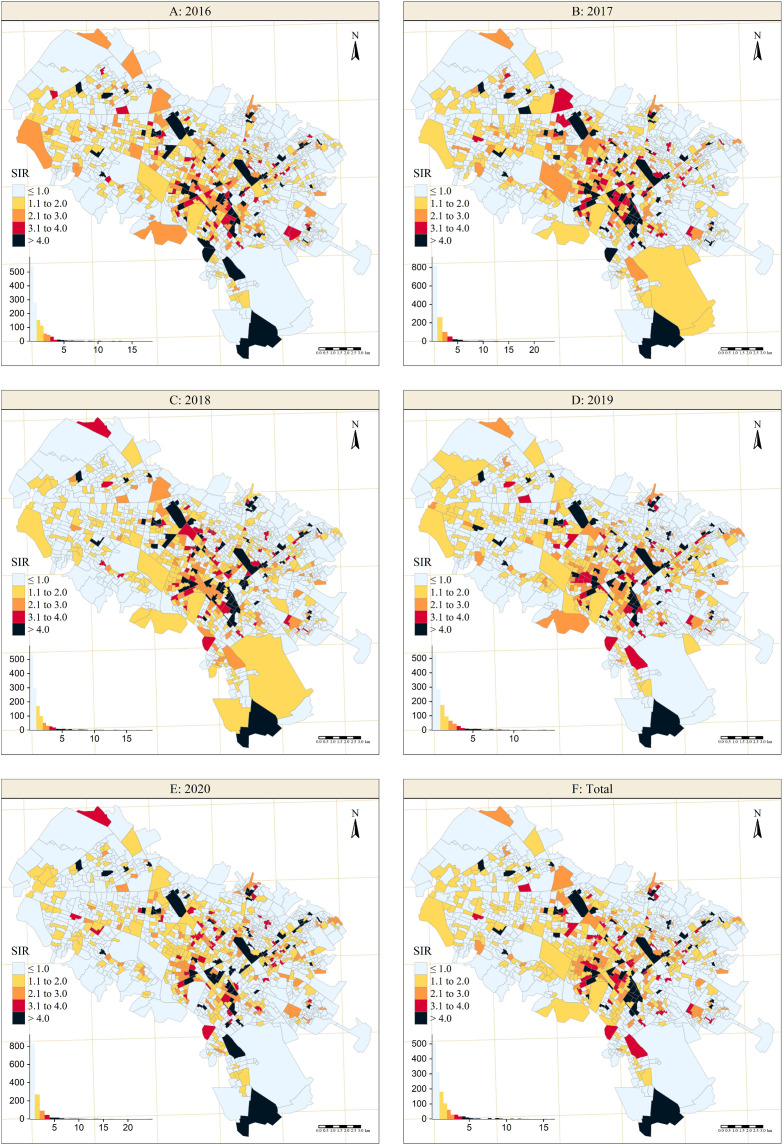
The annual spatial distribution of cardiovascular hospitalizations represented by the standardized incidence ratio (SIR) from 2016 to 2020. The accompanying histogram displays the number of census tracts on the y-axis and the SIR on the x-axis. Authors using R version 4.3.3 created maps.

Annual maps of standardized incidence ratios (SIRs) revealed persistent spatial heterogeneity across Mashhad from 2016 to 2020 (Fig 3). Census tracts with SIR > 1 were consistently concentrated in central and downtown areas, while lower-risk areas were more frequently observed in peripheral regions.

#### 3.3.2. Hot spot classification.

**Fig 4:**
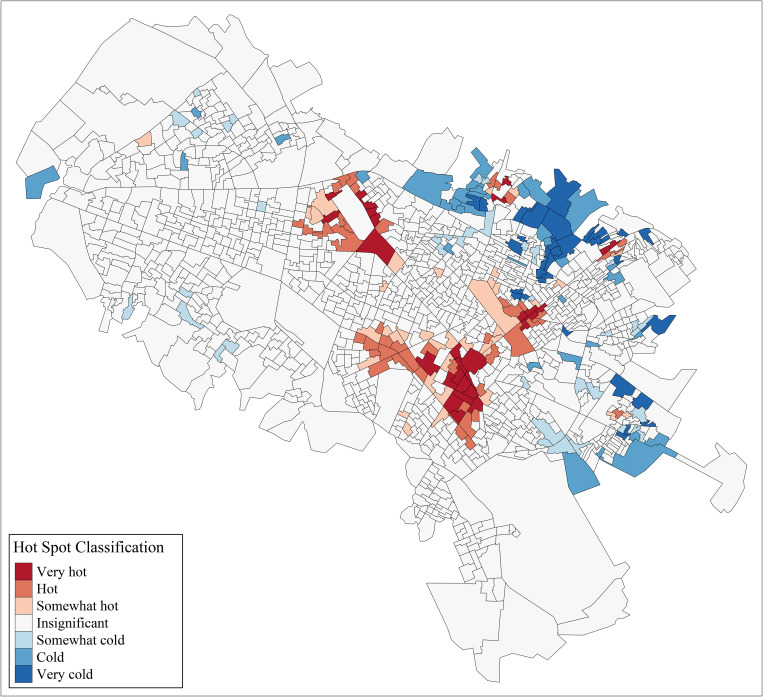
Hot and cold spot classifications of cardiovascular disease (CVD) hospitalization rates in the city of Mashhad, 2016-2020. Authors using R version 4.3.3 created the map.

Global spatial autocorrelation analysis yielded a Moran’s *I* of 0.176 (*p* < 0.001), indicating significant positive spatial clustering, meaning areas with similar values, either high or low, tend to cluster together. Hot spot analysis identified statistically significant high-rate clusters in central and downtown areas, whereas cold spots —indicating lower rates—were predominantly concentrated in the northern, northeastern, and eastern regions of the city (Fig 4).

#### 3.3.3. Flexible spatial scan clustering.

FSSS identified stable high-rate clusters in the city center across all five years. The initial SC location was consistent for the first four years, although the pattern of involved census tracts varied (Appendix 2). Conversely, in low-rate clusters, the MLC moved between two primary locations on the outskirts of the city (Appendix 3).

**Fig 5:**
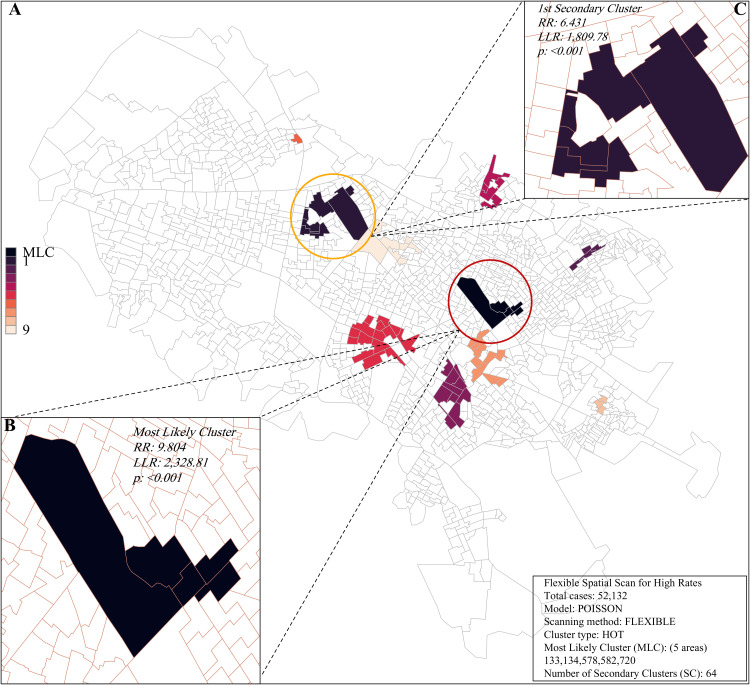
Flexible spatial scan analysis for high rates of cardiovascular disease (CVD) hospitalization in the city of Mashhad, 2016-2020; A: top 10 clusters in the entire study area; B: zoomed in on the most likely cluster (MLC); C: zoomed in on the first secondary cluster (SC). Authors using R version 4.3.3 created maps.

**Fig 6:**
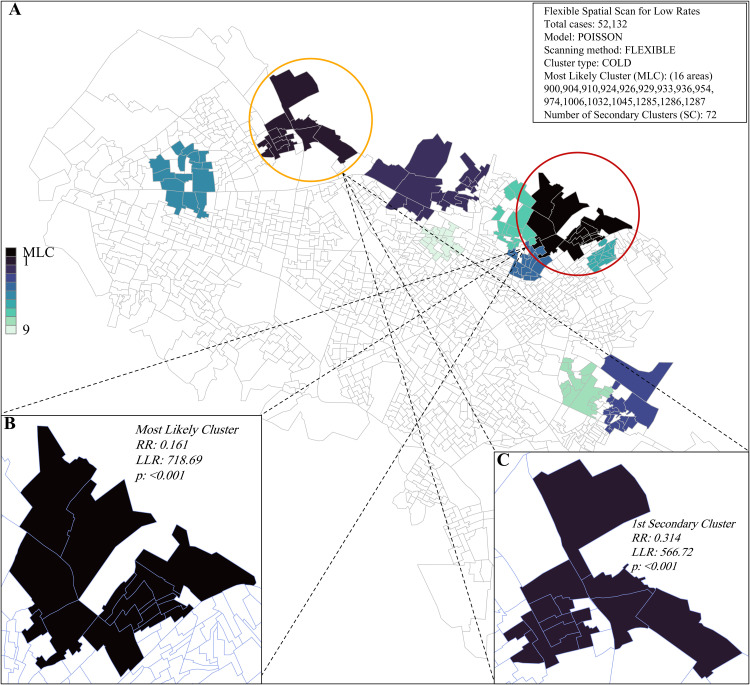
Flexible spatial scan analysis for low rates of cardiovascular disease (CVD) hospitalization in the city of Mashhad, 2016-2020; A: top 10 clusters in the entire study area; B: zoomed in on the most likely cluster (MLC); C: zoomed in on the first secondary cluster (SC). Authors using R version 4.3.3 created maps.

The MLC for high hospitalization rates exhibited an RR of 9.80 with an LLR of 2,328.81 (*p* < 0.001). Secondary high-rate clusters were primarily located in downtown areas (Fig 5; Table 3). Low-rate clusters were predominantly detected in eastern, northern, and northwestern peripheral regions. The MLC for low-rate clusters showed an RR of 0.16 and an LLR of 718.69 (*p* < 0.001) (Fig 6; Table 3). The location and composition of secondary low-rate clusters varied across years.

**Table 3:** Spatial scan statistics for overall high and low rates of cardiovascular disease (CVD) incidents using flexible spatial scanning method in the city of Mashhad, 2016-2020.

High-rate clusters (Hot)	N	Radius	Observed	Expected	RR	Stats (LLR)	*p*-Value
MLC	5	1,465	1,666	169.930	9.804	2,328.809	0.001
SC. 1	7	2,236	1,759	273.509	6.431	1,809.784	0.001
SC. 2	4	1,035	988	154.418	6.398	1,006.886	0.001
SC. 3	11	2,028	912	144.166	6.326	920.208	0.001
SC. 4	6	1,204	875	168.384	5.196	740.190	0.001
SC. 5	16	2,166	1,098	321.928	3.411	576.925	0.001
SC. 6	1	0	483	63.327	7.627	563.338	0.001
SC. 7	10	1,944	720	158.972	4.529	529.586	0.001
SC. 8	3	407	565	97.864	5.773	525.551	0.001
SC. 9	8	1,635	872	272.383	3.201	418.506	0.001
**Low-rate clusters (Cold)**							
MLC	16	2,718	208	1,295.611	0.161	718.690	0.001
SC. 1	14	2,707	538	1,714.715	0.314	566.722	0.001
SC. 2	17	3,585	211	1,020.809	0.207	483.551	0.001
SC. 3	15	2,251	113	774.056	0.146	447.851	0.001
SC. 4	18	1,293	126	681.015	0.185	345.398	0.001
SC. 5	15	2,306	302	991.649	0.305	335.218	0.001
SC. 6	14	1,148	57	515.051	0.111	334.608	0.001
SC. 7	13	1,998	58	450.531	0.129	275.119	0.001
SC. 8	13	1,902	110	513.556	0.214	235.632	0.001
SC. 9	16	1,350	254	762.628	0.333	231.881	0.001

MLC: Most Likely Cluster, SC: Secondary Cluster, N: Number of areas, RR: Relative Risk, LLR: Log-likelihood Ratio.

## 4. Discussion

This study identifies significant high-risk areas for cardiovascular hospitalization in Mashhad City. Analysis of a large dataset of confirmed CVD cases revealed an older median age, a concerningly high case fatality rate, and a predominance of male patients. Temporal analysis showed a sharp decline in CVD hospitalization during the early COVID-19 pandemic likely reflecting changes in healthcare-seeking behavior or service disruptions. Geographic mapping uncovered spatial disparities, with central and downtown areas showing higher CVD incidence and risk. These findings emphasize persistent high-risk zones, providing valuable insights for targeted interventions and resource allocation to address the uneven CVD burden in the region.

Ample epidemiological evidence indicates that age and gender significantly impact CVD risk [36]. Likewise, we found disease predominance among males and a concentration of cases among the elderly in Mashhad. This finding may be explained by smoking habits, a major risk factor for cardiac health [37]. In Iran, approximately 26% of males are smokers, which could contribute to the observed sex differences in cardiovascular risk [38]. As well, previous studies link male obesity in Iran to higher CVD risk, hypothesizing a similar pattern in this study [39].

Temporal trend investigation showed a clear decrease in CVD hospitalization during the early months of the COVID-19 pandemic. This aligns with the worldwide findings of lower healthcare use during that period [40,41], likely driven by changes in people’s behavior, with some evidence suggesting broader psychological impacts of the pandemic on daily activities [42]. However, the increase in CVD-related deaths during the second surge of the pandemic, despite fewer hospital admissions, indicates the pandemic’s indirect impact on health outcomes unrelated to direct COVID-19 infections. This finding aligns with previous research highlighting the indirect correlation of the pandemic and the increased mortality rates of chronic diseases during that time [43]. In this regard, the shortest LOS coincided with the onset of the COVID-19 pandemic, indicating that hospitals discharged patients more quickly to manage the surge.

The identified regional disparities in the burden of CVD hospitalization can be ascribed to a variety of environmental, socio-demographic, and behavioral determinants. The central and downtown areas of Mashhad, characterized by higher SIRs and identified as high-rate clusters, are marked by significant commercial activity, a densely packed population, and heavy vehicular traffic, all of which contribute to environmental and lifestyle factors that elevate the risk of CVD [12]. This formulation posits that the urban environment, with its bustling nature, may contribute to increased health risks. In contrast, the outskirts of the city identified more low-rate clusters, which suggests a potential correlation between lower CVD hospitalization rates and factors such as residential settings, reduced commercial development, lower levels of urban stressors, and potentially better access to green spaces that promote healthier lifestyles [12,21]. Differences between central and peripheral urban areas may reflect underlying socioeconomic and healthcare access factors that were not directly examined in this study. Central districts may exhibit higher hospitalization rates due to greater availability of specialized cardiovascular services and referral capacity, whereas peripheral areas may face barriers to healthcare access that influence hospitalization patterns. Economic conditions and related healthcare-seeking behaviors could also contribute to the observed spatial contrasts and warrant further investigation.

A recent review has demonstrated a beneficial association between green space and CVD [44]. Conversely, the built environment has been attributed to increased risks of CVD [45]. Our findings confirm these associations, as hotspots and high-rate clusters were primarily observed in the city’s central districts, which are marked by high commercial density and low green space. A study conducted in 2019 demonstrated that the presence of urban green spaces in Mashhad is spatially correlated with the incidence of CVD among women, indicating that access to these environments may significantly influence health outcomes [21]. This is supported by another study showing that public green spaces in central, densely populated districts have a lower ecological carrying capacity than those in the outskirts of Mashhad [46], suggesting reduced environmental quality and limited capacity to support health-promoting activities. Regarding the long-term effects of air pollution on CVD hospitalization [47,48], It can be hypothesized that the increase in urbanization and, consequently, air pollution in Mashhad over the past two decades [49] contributes to the onset of CVD and serves as a potential risk factor for the hotspot clusters concentrated in the city’s central business district.

This study represents the first extensive geographical analysis of cardiovascular hospitalization data over five years in Mashhad, providing detailed insights into where and when CVD hospitalizations are concentrated within the city (see our Data Note for a detailed description of the dataset and its structure [50]). While the analysis was not designed to establish causal links between CVD and specific environmental or socioeconomic exposures, it identifies priority areas for further investigation and targeted intervention. Future studies integrating individual-level socioeconomic, environmental, and behavioral data are warranted to elucidate the mechanisms underlying the observed spatial disparities.

### 4.1. Strengths and limitations

This study has several notable strengths. It is based on a large, population-level dataset covering more than 52,000 confirmed CVD hospitalizations over five consecutive years, enabling robust temporal and spatial analyses. The integration of time-series modeling, Joinpoint regression, and multiple spatial analytical techniques, including hotspot detection and flexible spatial scan statistics, provides a comprehensive and methodologically rigorous assessment of CVD hospitalization patterns. Additionally, the fine spatial resolution at the census-tract level enhances the ability to identify localized high-risk areas relevant for public health planning.

We acknowledge the limitations of our study. The reliance on hospital admission data may underestimate the true prevalence of CVD, as it does not account for patients treated in outpatient settings or those who do not seek care. Furthermore, the analysis period coincided with the COVID-19 pandemic, which may have affected CVD occurrence and outcomes, limiting the representativeness of long-term trends. Future research should include data from a wider range of healthcare settings and investigate the pandemic’s long-term impact on CVD epidemiology in the region.

## 5. Conclusions

In conclusion, this study reveals critical insights into the cardiovascular health landscape of Mashhad City, highlighting significant disparities in hospitalization rates tied to potential environmental, socio-demographic, and behavioral factors. The alarming concentration of CVD cases in high-risk areas, particularly within the bustling central districts characterized by low green space and high commercial density, underscores an urgent need for targeted public health interventions. Addressing these geographical disparities not only has the potential to improve cardiovascular outcomes but also promotes a healthier, more resilient community.

## Supporting information

S1 Appendix Inset maps of A) Iran and B) Razavi-Khorasan province, with the red area highlighting the location of Mashhad city. Authors using R version 4.3.3 created maps.
(JPEG)

S2 Appendix The annual flexible spatial scan analysis for the high-rate clusters of cardiovascular disease (CVD) from 2016 to 2020, with the red circle indicating the most likely cluster (MLC) and the blue circle representing the first secondary cluster. Authors using R version 4.3.3 created maps.
(PDF)

S3 Appendix The annual flexible spatial scan analysis for the low-rate clusters of cardiovascular disease (CVD) from 2016 to 2020, with the red circle indicating the most likely cluster (MLC) and the orange circle representing the first secondary cluster. Authors using R version 4.3.3 created maps.
(PDF)
