# Supplementary figures and images for: Cardiovascular hospitalization dynamics in Iran’s second-largest city: A spatial and temporal perspective
Source: PLoS One. 2026 Jul 6;21(7):e0352424. doi: 10.1371/journal.pone.0352424 (PMC13336199; doi:10.1371/journal.pone.0352424)

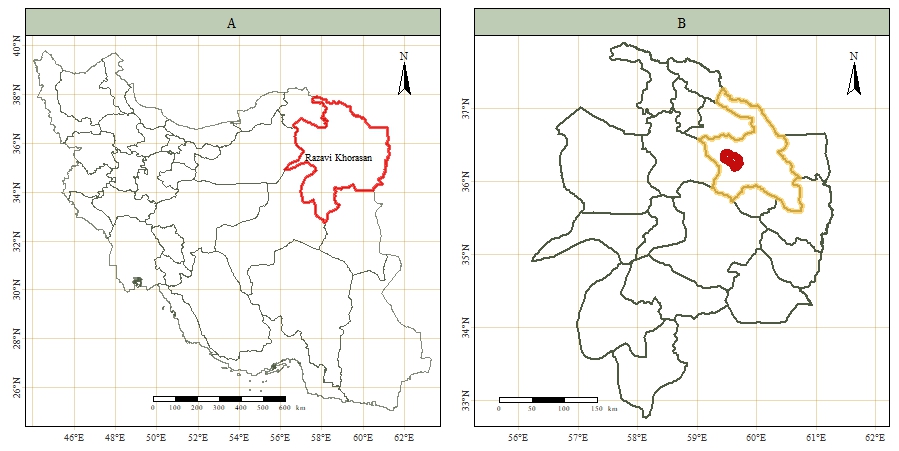

Supplement: S1 Appendix — Authors using R version 4.3.3 created maps. (JPEG) [file pone.0352424.s001.jpeg]

2016

HOT Clusters

- MLC
- SC.1
- SC.2
- SC.3
- SC.4
- SC.5
- SC.6
- SC.7
- SC.8
- SC.9

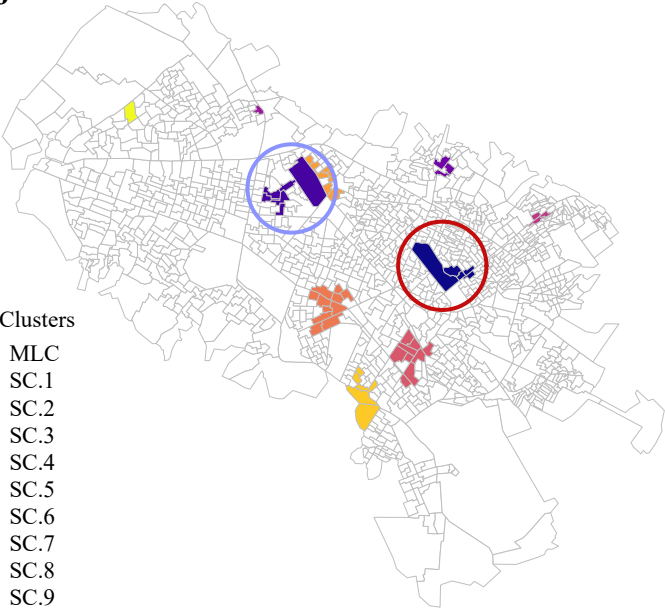

2017

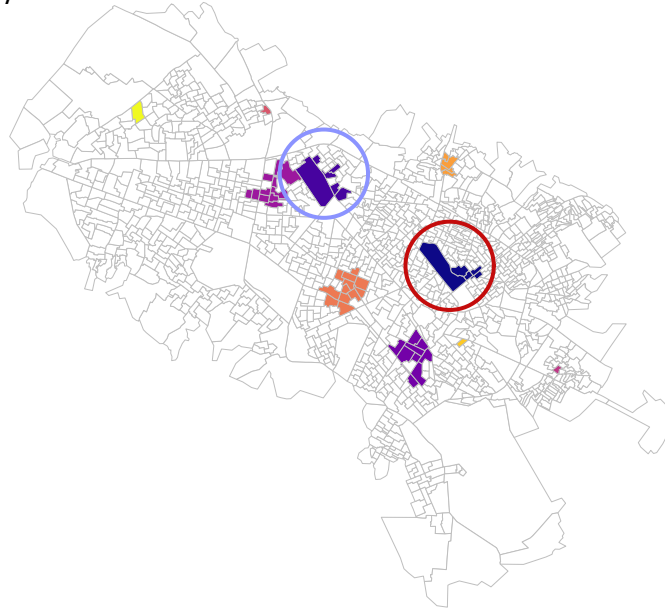

2018

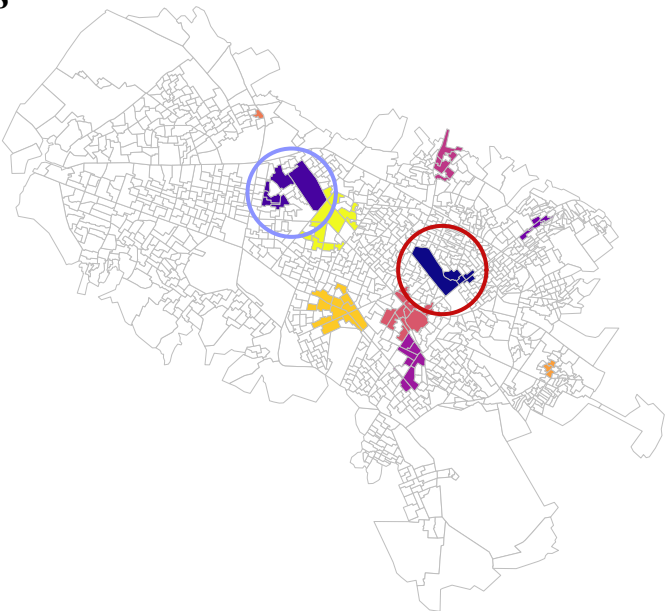

2019

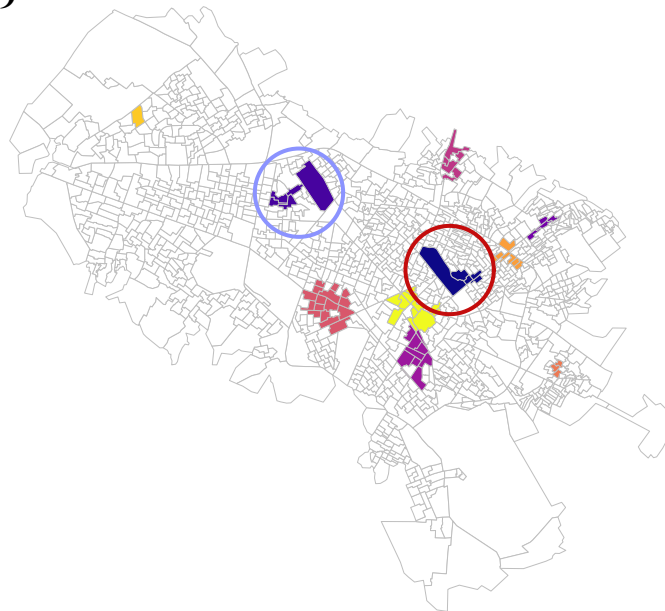

2020

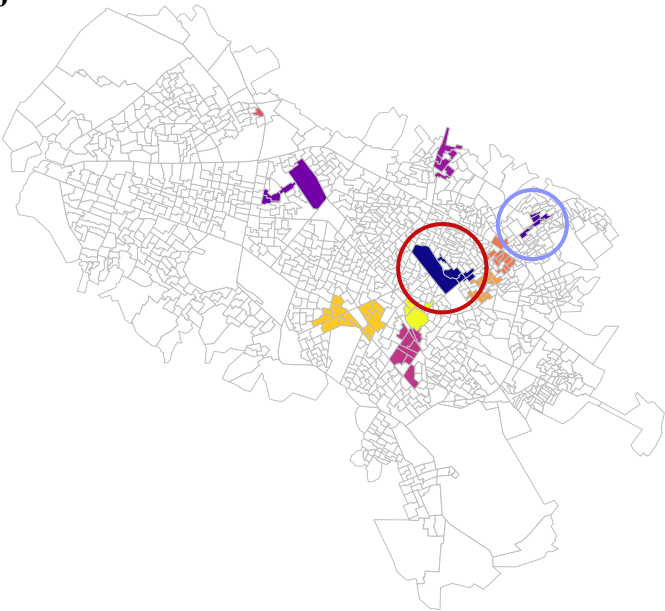

Total

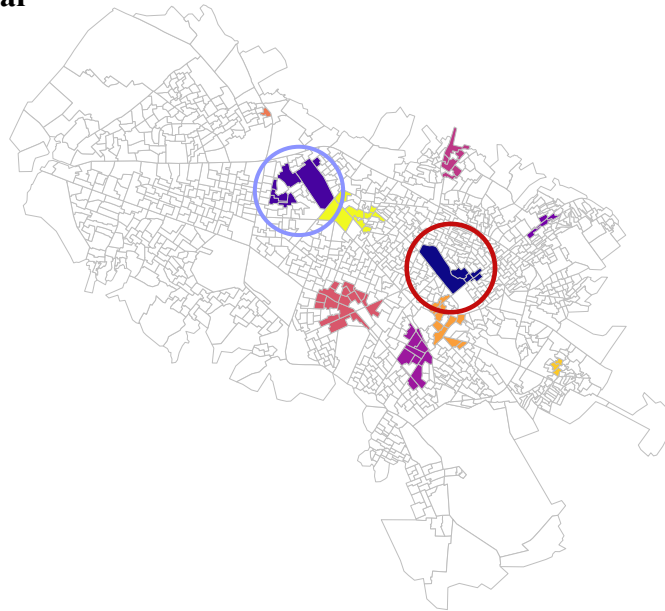

Supplement: S2 Appendix — Authors using R version 4.3.3 created maps. (PDF) [file pone.0352424.s002.pdf]

2016

COLD Clusters

- MLC
- SC.1
- SC.2
- SC.3
- SC.4
- SC.5
- SC.6
- SC.7
- SC.8
- SC.9

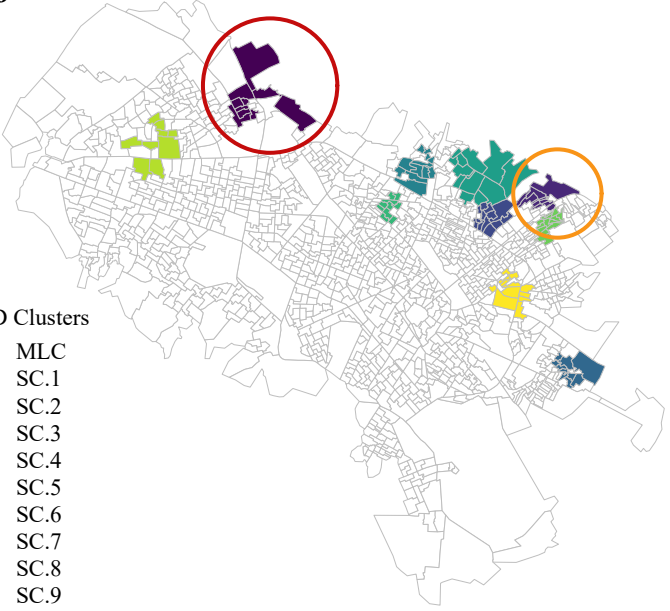

2017

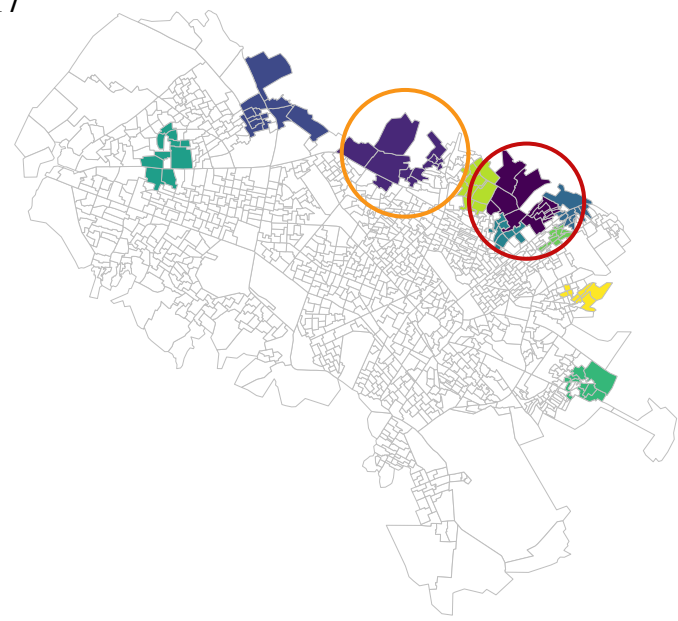

2018

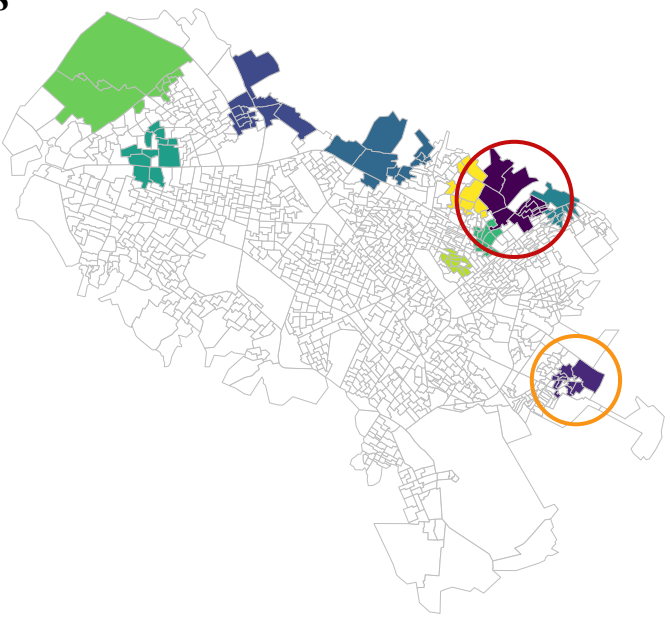

2019

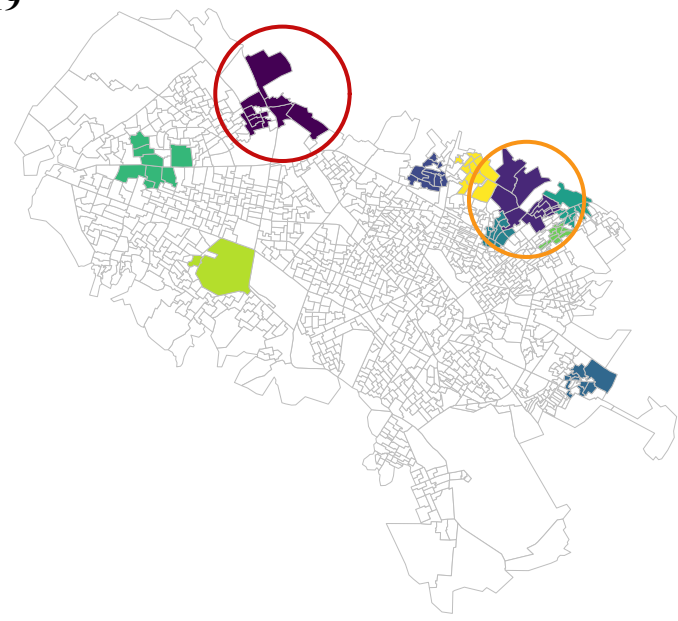

2020

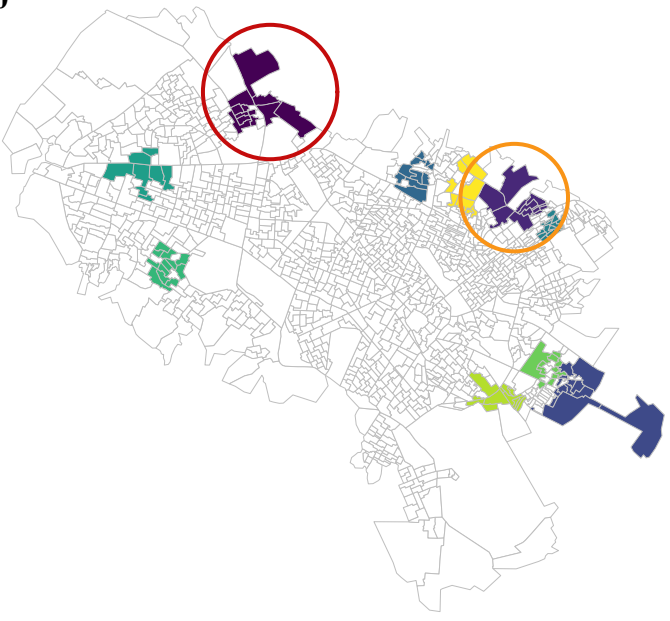

Total

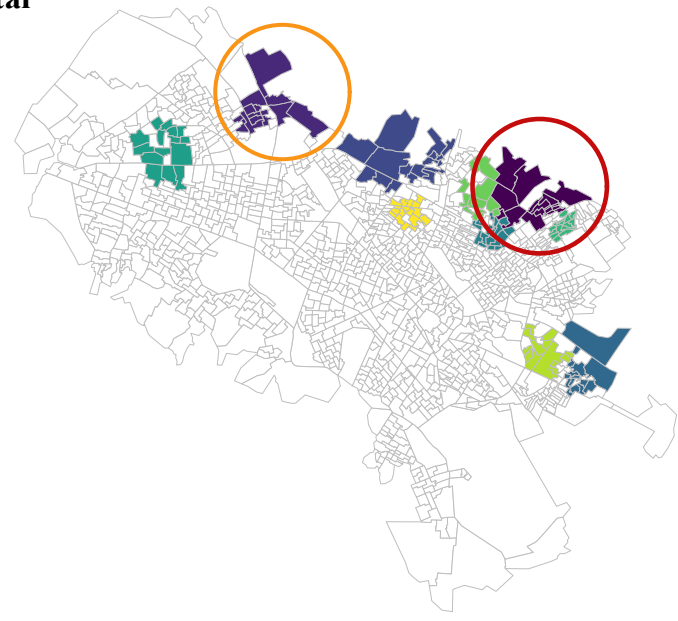

Supplement: S3 Appendix — Authors using R version 4.3.3 created maps. (PDF) [file pone.0352424.s003.pdf]
